# Delayed diagnosis of PRES and eclampsia in a concealed pregnancy

**DOI:** 10.11604/pamj.2014.19.299.5628

**Published:** 2014-11-18

**Authors:** Zeynep Ozcan Dag, Yavuz Simsek, Yakup Turkel, Ozlem Banu Tulmac, Yuksel Isik

**Affiliations:** 1 Department of Obstetrics and Gynecology, Kirikkale University, Faculty of Medicine, Kirikkale, Turkey; 2 Department of Neurology, Kirikkale University, Faculty of Medicine, Kirikkale, Turkey

**Keywords:** Concealed pregnancy, eclampsia, posterior reversible encephalopathy syndrome, pre-eclampsia

## Abstract

Pre-eclampsia and eclampsia are well-known risk factors of posterior reversible encephalopathy syndrome. Early recognition and proper treatment result in complete reversibility of this disease. Concealed pregnancy obstacles a safe prenatal care and a safe planned delivery, because of latency in the diagnosis. We present a case of unrecognized posterior reversible encephalopathy syndrome, eclampsia and premature delivery due to concealed pregnancy.

## Introduction

Preeclampsia (PE) remains a leading cause of maternal and perinatal mortality and morbidity. The rate of incidence varies upon the study population but generally ranges from 3% to 7% of all pregnancies [1]. When PE remains untreated, it moves towards a more serious condition known as eclampsia, Eclampsia is defined by the presence of seizures [2]. Pre-eclampsia and eclampsia are both well-known risk factors of posterior reversible encephalopathy syndrome (PRES). Early recognition and proper treatment result in complete reversibility of this disease. The risk of maternal and fetal mortality and morbidity increase in concealed pregnancies due to poor prenatal care [3]. We present a case of unrecognized posterior reversible encephalopathy syndrome, eclampsia and premature delivery due to concealed pregnancy.

## Patient and observation

**Figure 1:**
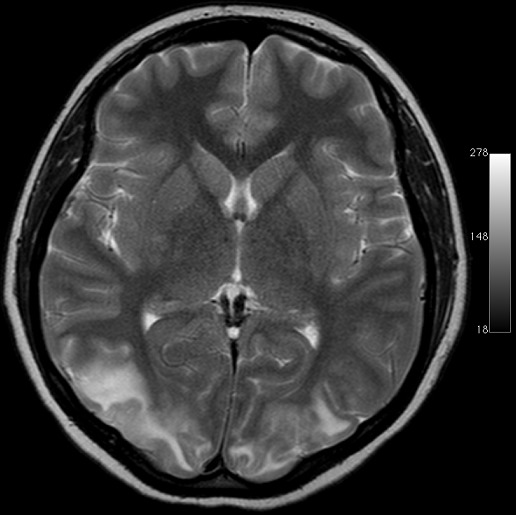
T2WI MRI of patient

A 25 years old, multiparaous widow female patient with loss of consciousness and generalyzed convulsion was admitted to a hospital. She was hospitalized for the diagnosis and treatment. Her arterial blood pressure was 180/100 mmHg on admission. Hypoalbunemia and anemia were detected in her laboratory findings and she was given albumin and erythrocyte suspensions. Amlodipine 10 mg/day was started for hypertension. The patient was referred to us for further examination and treatment. She was concious when admitted to the emergency clinic. The patient was hospitalized by the neurology clinic with a diagnosis of hypertensive encephalopathy. Hyperintensity in bilateral parieto-occipital region and was observed in magnetic resonance imaging that was compatible with PRES (Figure 1). Initial medical history did not reveal a delayed menstruation or pregnancy. The laboratory findings showed serum aspartate aminotransaminase (AST): 115 U/L (N: 0-35), serum alanine aminotransaminase (ALT): 26 IU/L (N: 0-45), serum urea and creatine to be normal, white blood cell (WBC): 6100/mm^3^ (N: 4400-11300), hemoglobin (Hb): 10.5mg/dL (N: 11.5-16.0), and platelet count (Plt): 165.000/mm^3^ (N:150.000-;450000) and a catheterized urine specimen demonstrated proteinuria (+ + +). To investigate the etiology of hypertension, renal ultrasound examination was performed. During ultrasonography a live fetus in the uterine cavity was detected. She was admitted to the Neurology Department with an initial diagnosis hypertension, eclampsia and HELLP. There was a live fetus with olygohydroamnios compatible with 21 weeks of gestation in her obstetrical ultrasonography. Arterial blood pressure was 150/90 and repeated laboratory findings revealed serum aspartate aminotransaminase (AST): 66 U/L (N: 0-35), serum alanine aminotransaminase (ALT): 25 IU/L (N: 0-45), serum lactate dehydrogenase (LDH): 359 IU/L (N: 25-247), serum urea and creatine to be normal, white blood cell (WBC): 5700/mm^3^ (N: 4400-11300), hemoglobin (Hb): 9.7mg/dL (N: 11.5-16.0), and platelet count (Plt): 118.000/mm^3^ (N: 150.000-450000) and a catheterized urine specimen demonstrated proteinuria (+ + ). She stated that she concealed the pregnancy as it was out of wedlock. She was G2P1, and she did not remember the last menstruel period. No seizures took place after hospitalization. Arterial blood pressure was controlled with antihypertensive (alpha metyl dopa). Magnesium sulphate was started. After 24-hours, urine protein increased to 5791 mg, arterial blood pressure elevated (160/100) and due to the onset of symptoms suggestive of severe preeclampsia such as olyguria, visual symptoms, upper abdominal pain, she was taken to operating room for an emergency cesarean section and one alive female baby in vertex presentation was delivered. With these findings, the patient was diagnosed as having HELLP syndrome and eclampsia. Although latency in the diagnosis due to concealment of pregnancy, she was treated successfully and unfortunately, the fetus died in the third day of delivery.

## Discussion

PRES is a clinicoradiologic entity characterized by headaches, altered mental status, seizures, and visual loss; it is associated with white matter vasogenic edema predominantly affecting the occipital and parietal lobes of the brain. The most characteristic imaging pattern in PRES is the presence of edema involving the white matter of the posterior portions of both cerebral hemispheres, especially the parietooccipital regions, in a relatively symmetric pattern that spares the calcarine and paramedian parts of the occipital lobes.[4–6]. Although the pathogenesis of PRES and imaging appearances are similar, the clinical syndrome can be variable with symptoms ranging from mild blurring of vision to severe neurological deficit. Our patient had noted altered sensorium and seizures. No focal neurological deficits and visual loss was observed. Ophthalmic examination did not reveal any abnormalities. The most common finding noted on MRI included bilateral symmetrical hyperintensities on T2-weighted images and fluid attenuated inversion recovery (FLAIR) sequences in the parieto-occipital regions [7]. The cause of PRES is not yet understood. Several factors can trigger the syndrome, most commonly: acute elevation of blood pressure, abnormal renal function, eclampsia, transplantation, neoplasia and chemotherapy treatment, systemic infections, renal disease acute or chronic [8]. Eclampsia is one of the most common situations described in association with PRES. Our patient had acute elevation of blood pressure and eclampsia which might have triggered the syndrome. The current criteria for diagnosis of PE are systolic blood pressure (SBP) ≥140 mm Hg or diastolic blood pressure (DBP) ≥90 mm Hg with proteinuria of ≥0.3 g/day [9]. Clinical and laboratory tests are intended to define and determine the severity of PE. Headaches, tinnitus, visual disorders, brisk tendon reflexes, and vigilance disorders are related to cerebral edema; oliguria to acute renal failure; uterine contraction, vaginal bleeding to placental abruption; vomiting to HELLP syndrome (Hemolysis Elevated Liver enzymes Low Plaquet count); bandlike epigastric pain to subcapsular hepatic hematoma; and dyspnea to cardiac failure. Although she had hypoalbuminemia and hypertension, pregnancy and pre-eclampsia were not considered in the differential dignosis. The patient was treated for hypertension and she received albumin replacement. To investigate the etiology of convulsions and loss of consciousness she was exposed to X-ray. She concealed the pregnancy as her pregnancy was out of wedlock and feared of a possible family violence. Thus the diagnosis was delayed. Finally, during the investigations, pregnancy was realized in the renal ultrasonography. Hide of pregnancy has been implicated in potentially jeopardising prenatal care and subsequent safe planned deliveries. Nirmal et al. have founded that prematurity rates were significantly higher in the concealed pregnancy cohort. Despite the low incidence of maternal morbidity, these women should be regarded as high-risk labour due to the increased perinatal morbidity [3]. There is a maternal and fetal death due to a concealed pregnancy with placenta previa in literature [10].

In clinical practice, it is common that a pregnancy may remain unrecognized up to the end of the first trimester, especially for primiparous women who are unfamiliar with the symptoms of pregnancy [1]. However, from the point of view of obstetric practice, a pregnancy that remains un-booked in the second and third trimester is considered highly unusual and can pose a severe threat to the life and health of the child and mother involved [2]. Newborns following denied pregnancies are delivered after either late onset or total absence of antenatal care, with a presumed subsequently increased risk for neonatal outcome. For this specific group, several characteristic outcome parameters were investigated. The data underline the elevated fetal outcome risk for newborns after denial of pregnancy. In this group, total absence or late onset of antenatal care results in a manifestation of pregnancy dependent risks. Preterm births and small for gestational age newborns, together with deaths, may be classified as at least potentially avoidable. Eclampsia, the major neurological complication of pre-eclampsia, is defined as a convulsive episode or any other sign of altered consciousness arising in a setting of PE, and which cannot be attributed to a pre-existing neurological condition. Delivery is the only curative treatment for PE [11].

## Conclusion

A concealed pregnancy may lead to both maternal and fetal death. Hypertension, convulsions, and proteinuria should suggest a diagnosis of eclampsia in a young woman. And in a case of suspected pregnancy, an initial ultrasonographic examination should be performed prior to X-ray.

## References

[1] Steegers EAP, von Dadelszen P, Duvekot JJ, Pijnenborg R (2010). Pre-eclampsia. Lancet..

[2] Duckitt K, Harrington D (2005). Risk factors for pre-eclampsia at antenatal booking: systematic review of controlled studies. BMJ..

[3] Nirmal D, Thijs I, Bethel J, Bhal PS (2006). The incidence and outcome of concealed pregnancies among hospital deliveries: an 11-year population-based study in South Glamorgan. J Obstet Gynaecol..

[4] Hinchey J, Chaves C, Appignani B, Breen J, Pao L, Wang A, Pessin MS, Lamy C, Mas JL, Caplan LR (1996). A reversible posterior leukoencephalopathy syndrome. N Engl J Med..

[5] Schwartz RB (2002). Hyperperfusion encephalopathies: hypertensive encephalopathy and related conditions. Neurologist..

[6] Bartynski WS, Boardman JF (2008). Catheter angiography, MR angiography, and MR perfusion in posterior reversible encephalopathy syndrome. Am J Neuroradiol..

[7] Karuppannasamy D, Vikrant K, Raghuram A, Kumaar TM (2014). Cortical visual loss in posterior reversible encephalopathy syndrome in late postpartum eclampsia: case series. Indian J Ophthalmol..

[8] Marrone LC, Gadonski G, Diogo LP, Brunelli JP, Martins WA, Laguna Gde O, Bahlis LF, Filho JR, da Costa BE, Poli-de-Figueiredo CE, Marrone AC, da Costa JC (2014). Posterior reversible encephalopathy syndrome: differences between pregnant and non-pregnant patients. Neurol Int..

[9] Steegers EAP, von Dadelszen P, Duvekot JJ, Pijnenborg R (2010). Pre-eclampsia. Lancet..

[10] Jovanovic B, Dordevic M (2006). Maternal and fetal death due to placenta previa/accreta in a concealed pregnancy-a case report. Med Pregl..

[11] Uzan J, Carbonnel M, Piconne O, Asmar R, Ayoubi JM (2011). Preeclampsia: pathophysiology, diagnosis, and management. Vasc Health Risk Manag..

